# Supplementary material for: Genome-wide association study of REM sleep behavior disorder identifies polygenic risk and brain expression effects
Source: Nat Commun. 2022 Dec 5;13:7496. doi: 10.1038/s41467-022-34732-5 (PMC9722930; doi:10.1038/s41467-022-34732-5)
Supplement: Supplementary file 1 — Supplementary Information [file 41467_2022_34732_MOESM1_ESM.pdf]

SUPPLEMENTARY MATERIALS: Genome-wide association study of REM sleep behavior disorder identifies polygenic risk and brain expression effects

TABLE OF CONTENTS

Supplementary Figures **1**

Figure S1: QQ plots for each cohort and the meta-analysis. *1*

Figure S2: LocusZoom regional Manhattan plots for GWAS nominated RBD risk loci *2*

Figure S3: Conditional analysis LocusZoom plots for the SCARB2 locus *3*

Figure S4: Association of RBD risk with SNCA-AS1 expression *4*

Figure S5: Sensitivity analysis of SNCA-AS1 colocalization *5*

Figure S6: Sensitivity analysis of MMRN1 colocalization *6*

Figure S7: Regional association plot for eQTL and RBD GWAS colocalization in the region surrounding SCARB2 *7*

Figure S8: Sensitivity analysis of SCARB2 colocalization using eQTLGen-derived eQTLs *8*

Figure S9: Sensitivity analysis of SCARB2 colocalization using PsychENCODE-derived eQTLs *9*

Figure S10: Comparing direction of effect for synucleinopathy loci and PD AAO *10*

Figure S11: Power calculations for the meta-analysis *11*

Supplementary Tables **12**

Table S1: Replication of meta-analysis variants in an independent PD+pRBD cohort *12*

Table S2: Directly genotyped rare variants with previously reported relationships with RBD *12*

SUPPLEMENTARY FIGURES

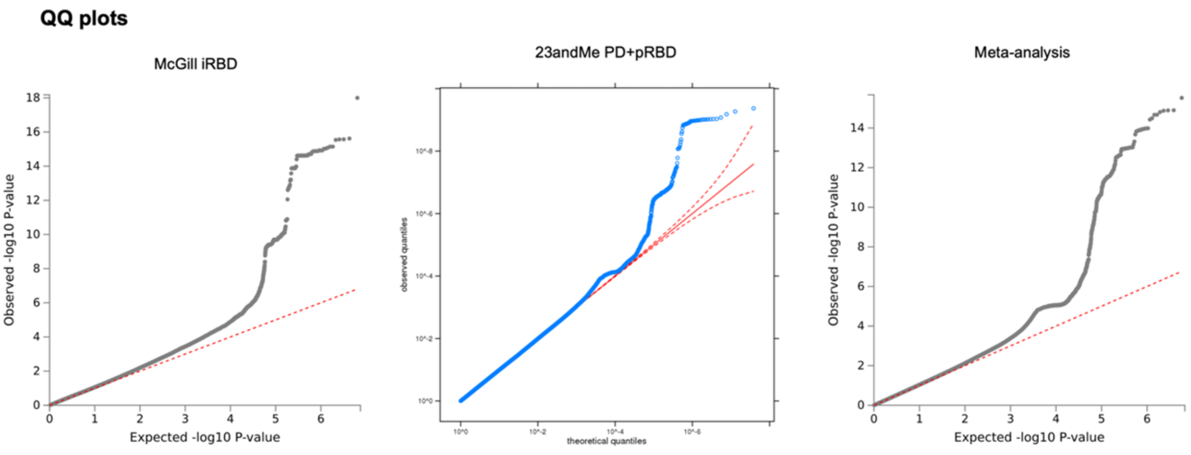

Supplementary Figure 1. QQ-plots for each cohort and the meta-analysis. The points represent the log-adjusted two-sided p-values from genome-wide association study, repeated logistic regression across the genome, adjusted for age, sex, and principal components. Plots for the McGill iRBD cohort and the meta-analysis were generated by FUMA (https://fuma.ctglab.nl/).

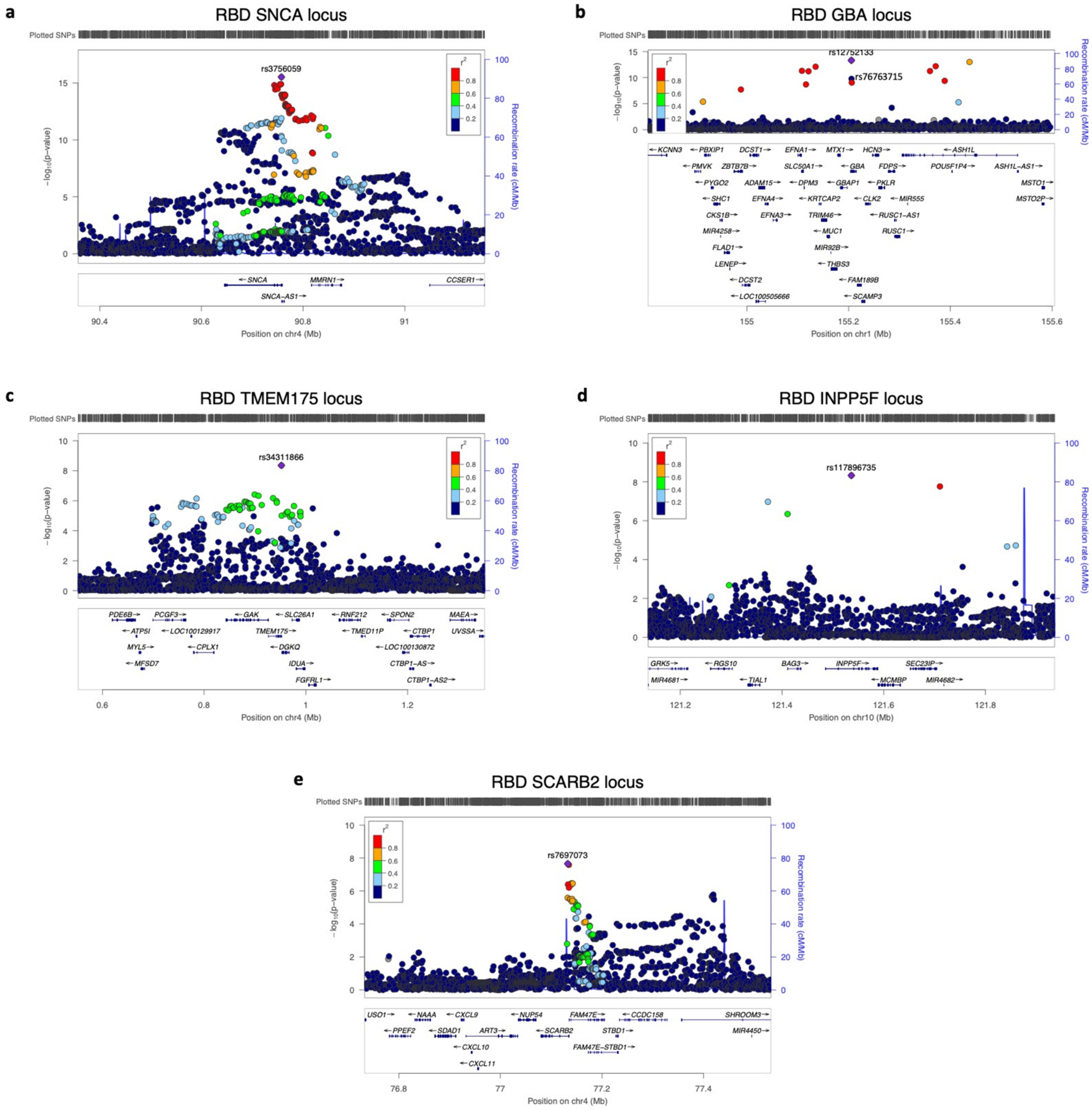

Supplementary Figure 2. LocusZoom regional Manhattan plots for GWAS nominated RBD risk loci. The points represent the log-adjusted two-sided p-values from genome-wide association study, repeated logistic regression across the genome, adjusted for age, sex, and principal components. Plots were generated using LocusZoom (http://locuszoom.org/). The x-axis indicates the genomic region defined by the top hit +/- 400kb, while the y axis represents the log-adjusted p values.

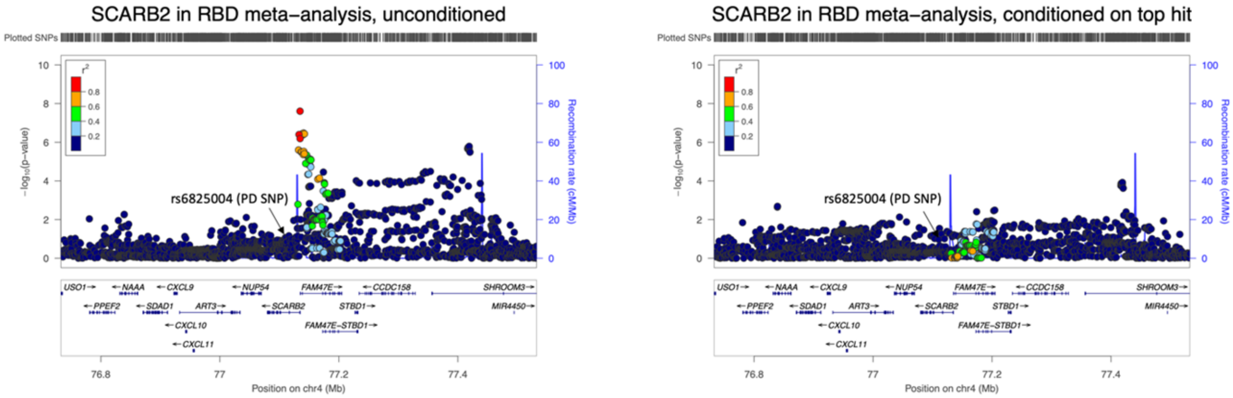

Supplementary Figure 3. LocusZoom regional Manhattan plots for the RBD meta-analysis results at the *SCARB2* locus, unconditioned (left) and conditioned on the top signal (right) using conditional-joint analysis via GCTA-COJO. The x-axis indicates the genomic region defined by the top hit +/- 400kb, while the y axis represents the log-adjusted two-sided p-values from genome-wide association study (GWAS), repeated logistic regression across the genome, adjusted for age, sex, and principal components. The conditioned plot (right) is also adjusted for the top signal from the unadjusted analysis. The GWAS-nominated PD variant, rs6825004, is labeled to show its lack of significance in this RBD analysis. Plots were generated using LocusZoom (http://locuszoom.org/). Colors indicate the strength of linkage-disequilibrium between points (SNPs).

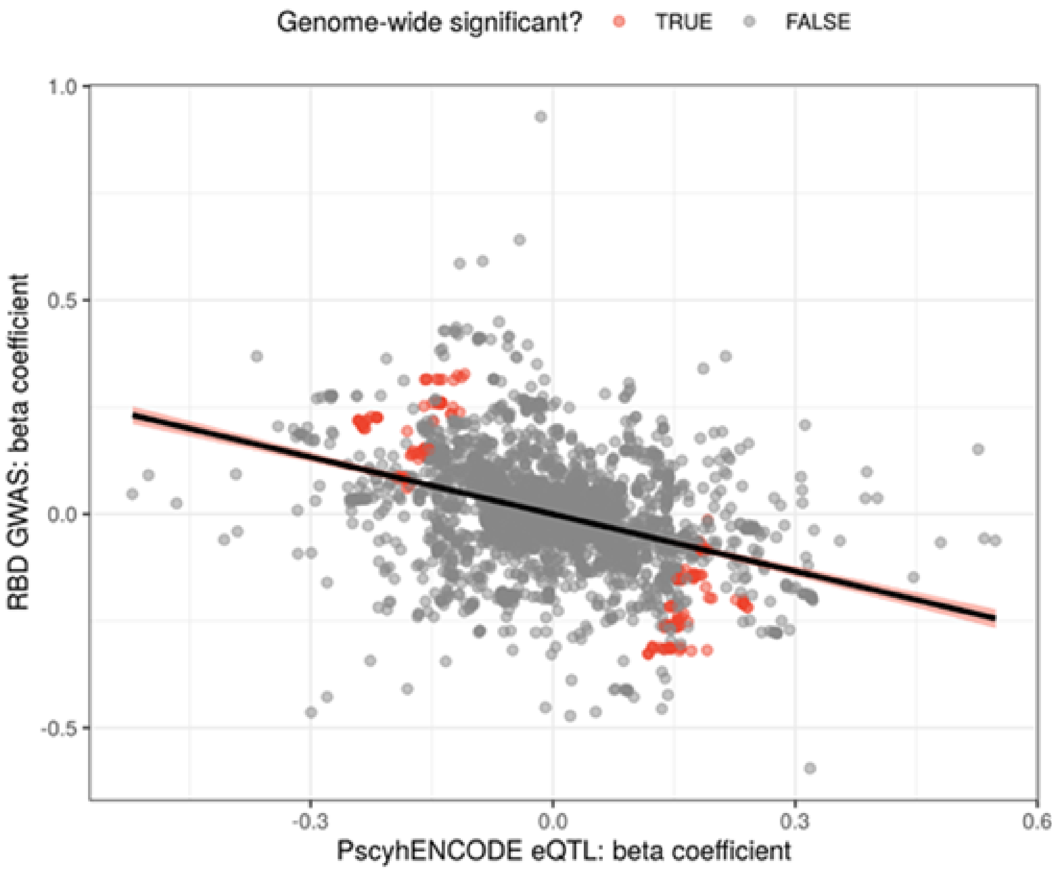

Supplementary Figure 4. Association of RBD risk with *SNCA-AS1* expression. Scatterplot of beta coefficients for SNPs shared between the RBD GWAS (logistic regression across the genome) and PsychENCODE eQTLs (linear regression) regulating *SNCA-AS1* expression. SNPs passing genome-wide significance (two-sided $p = 5 \times 10^{-8}$) in the RBD GWAS and/or PyschENCODE are indicated in red. The black line represents a linear model fitted for the beta coefficients from either dataset, with the 99% confidence interval indicated with a red fill.

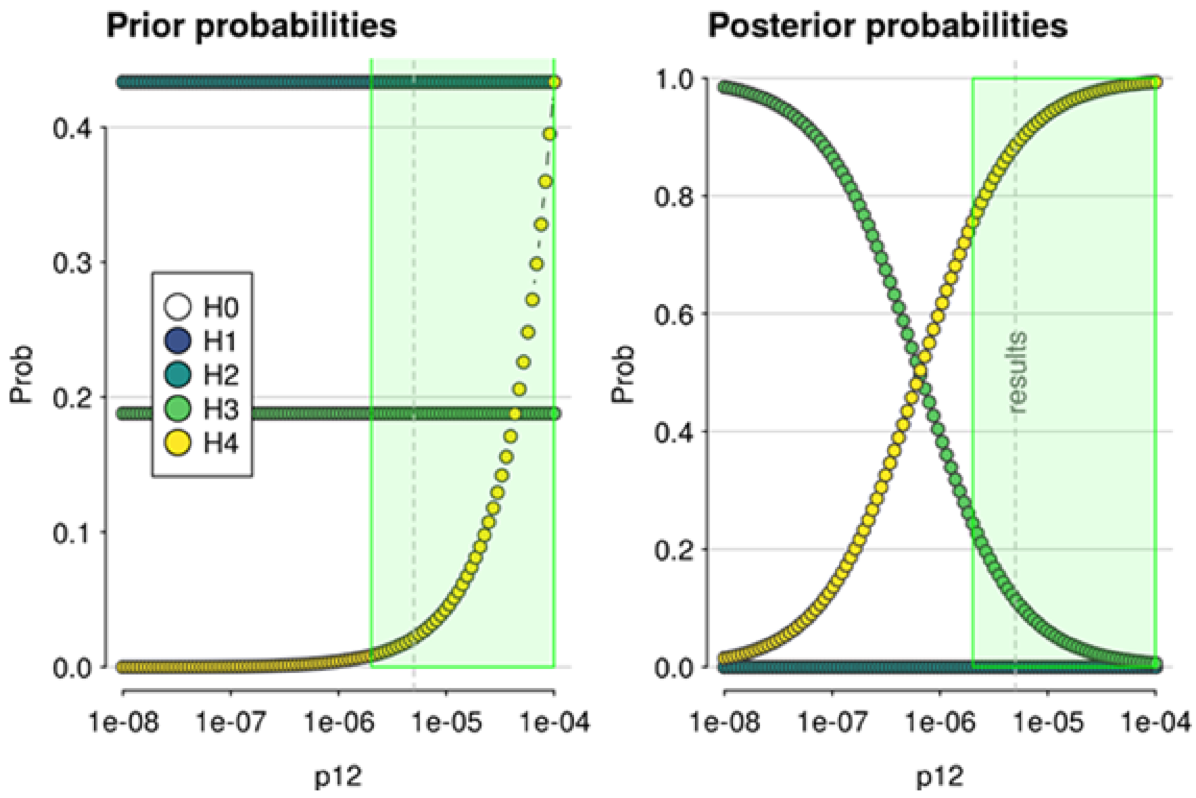

Supplementary Figure 5. Sensitivity analysis of *SNCA-AS1* colocalization. Sensitivity analysis of colocalization between PscyhENCODE-derived eQTLs regulating *SNCA-AS1* expression and RBD GWAS signals was performed using coloc sensitivity() function. Plot of prior (left) and posterior (right) probabilities for H0-H4 across varying p_{12} priors. Dashed vertical line indicates the value of p_{12} used in the initial analysis ($p_{12} = 5 \times 10^{-6}$). The green region in these plots show the region for which PPH4 ≥ 0.75 would still be supported.

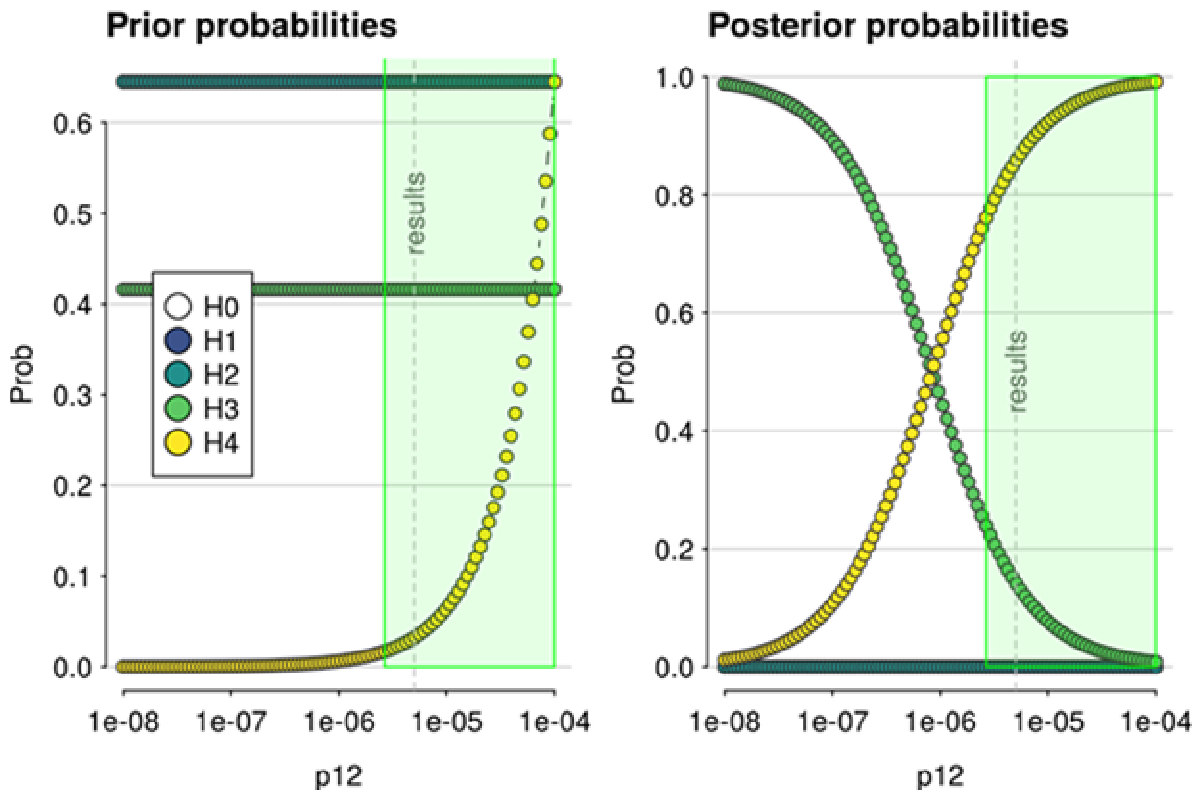

Supplementary Figure 6. Sensitivity analysis of *MMRN1* colocalization. Sensitivity analysis of colocalization between eQTLGen-derived eQTLs regulating MMRN1 expression and RBD GWAS signals was performed using coloc sensitivity() function.. Plot of prior (left) and posterior (right) probabilities for H0-H4 across varying p_{12} priors. Dashed vertical line indicates the value of p_{12} used in the initial analysis ($p_{12} = 5 \times 10^{-6}$). The green region in these plots show the region for which PPH4 ≥ 0.75 would still be supported.

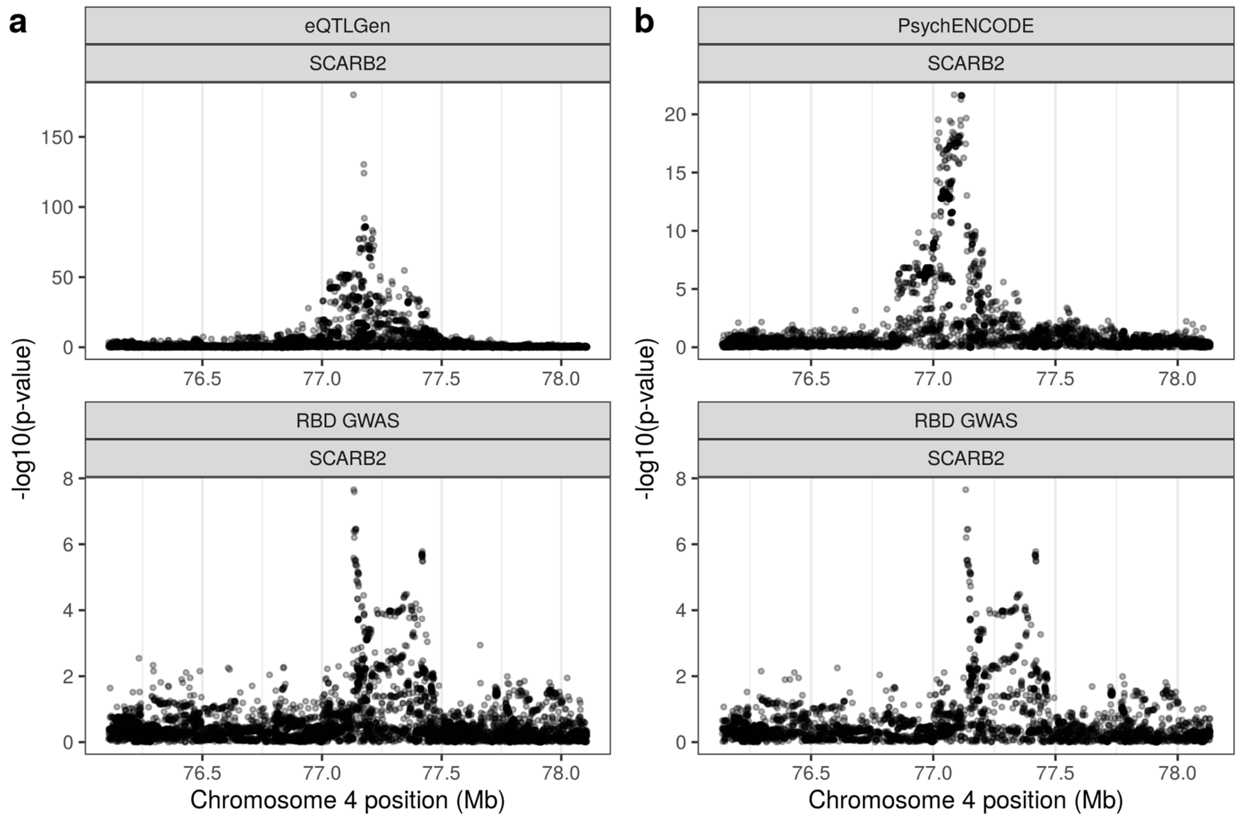

Supplementary Figure 7. Regional association plot for eQTL and RBD GWAS colocalization in the region surrounding *SCARB2*. Regional association plots for eQTL (upper pane) and RBD GWAS association signals (lower pane) in the region surrounding *SCARB2*, using eQTLs derived from (a) the eQTLGen meta-analysis of 31,684 blood samples from 37 cohorts (PPH3 = 0.99; PPH4 = 0.01) or (b) PsychENCODE's analysis of adult brain tissue from 1387 individuals (PPH3 = 0.66; PPH4 = 0.33). The x-axis denotes chromosomal position in hg19, and the y-axis indicates association p-values on a $-\log_{10}$ scale.

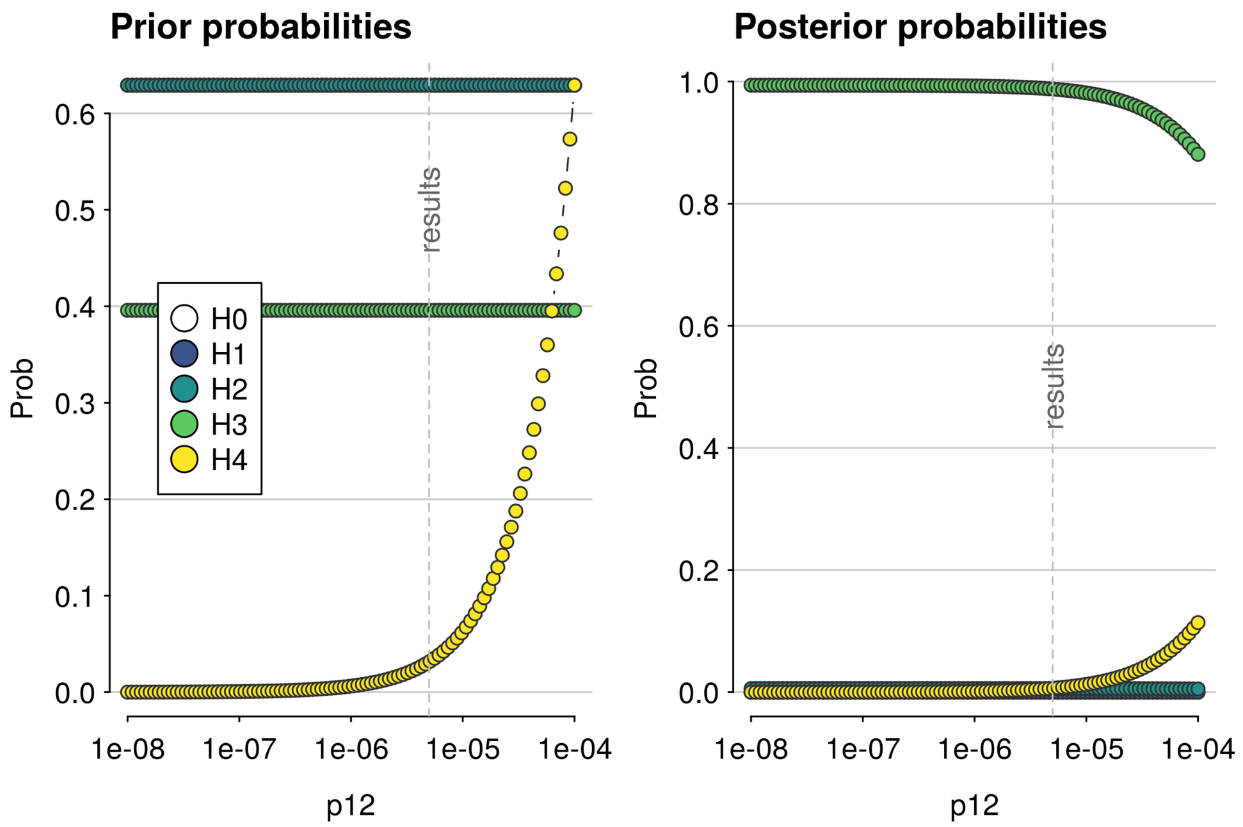

Supplementary Figure 8. Sensitivity analysis of *SCARB2* colocalization using eQTLGen-derived eQTLs. Sensitivity analysis of colocalization between eQTLGen-derived eQTLs regulating *SCARB2* expression and RBD GWAS signals. Plot of prior (left) and posterior (right) probabilities for H0-H4 across varying p_{12} priors. Dashed vertical line indicates the value of p_{12} used in the initial analysis ($p_{12} = 5 \times 10^{-6}$). The green region in these plots show the region for which PPH4 $\geq$ 0.75 would still be supported.

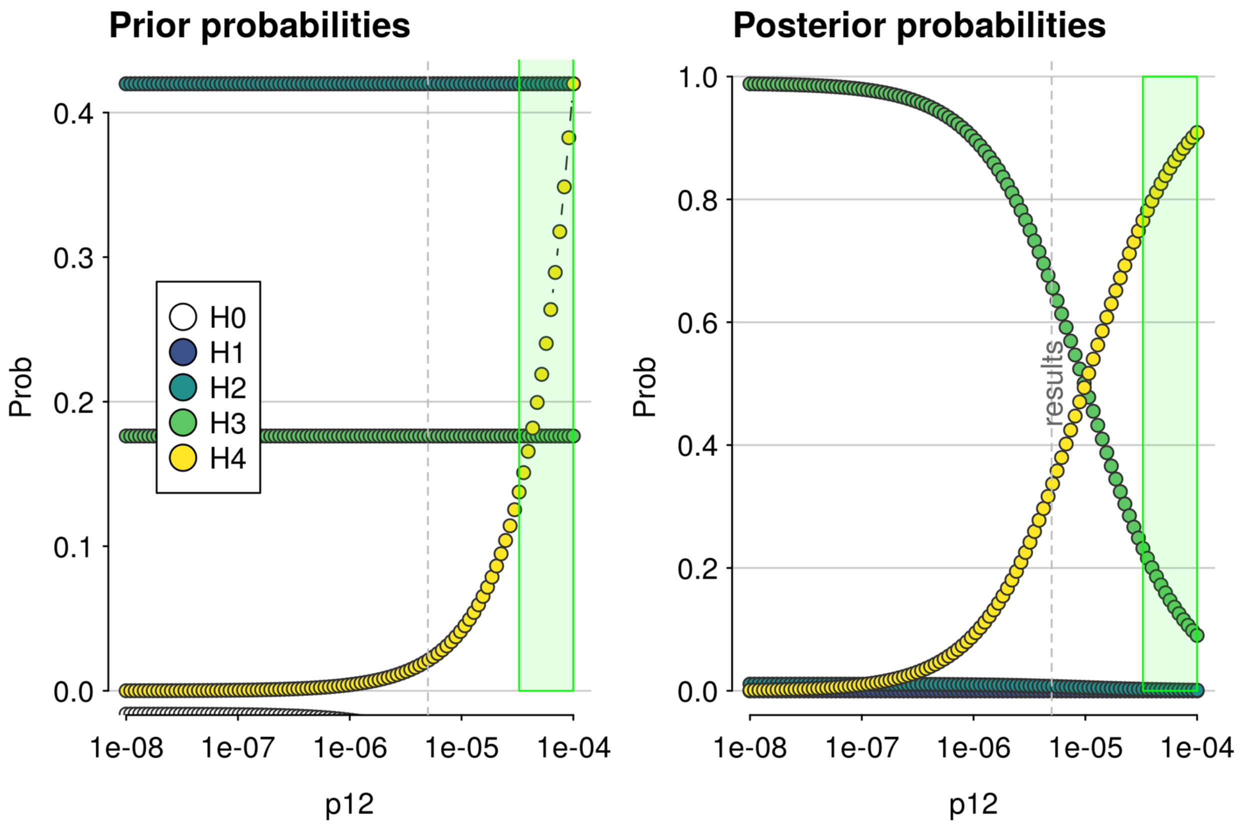

Supplementary Figure 9. Sensitivity analysis of *SCARB2* colocalization using PsychENCODE-derived eQTLs. Sensitivity analysis of colocalization between PsychENCODE-derived eQTLs regulating *SCARB2* expression and RBD GWAS signals. Plot of prior (left) and posterior (right) probabilities for H0-H4 across varying p_{12} priors. Dashed vertical line indicates the value of p_{12} used in the initial analysis ($p_{12} = 5 \times 10^{-6}$). The green region in these plots show the region for which PPH4 ≥ 0.75 would still be supported.

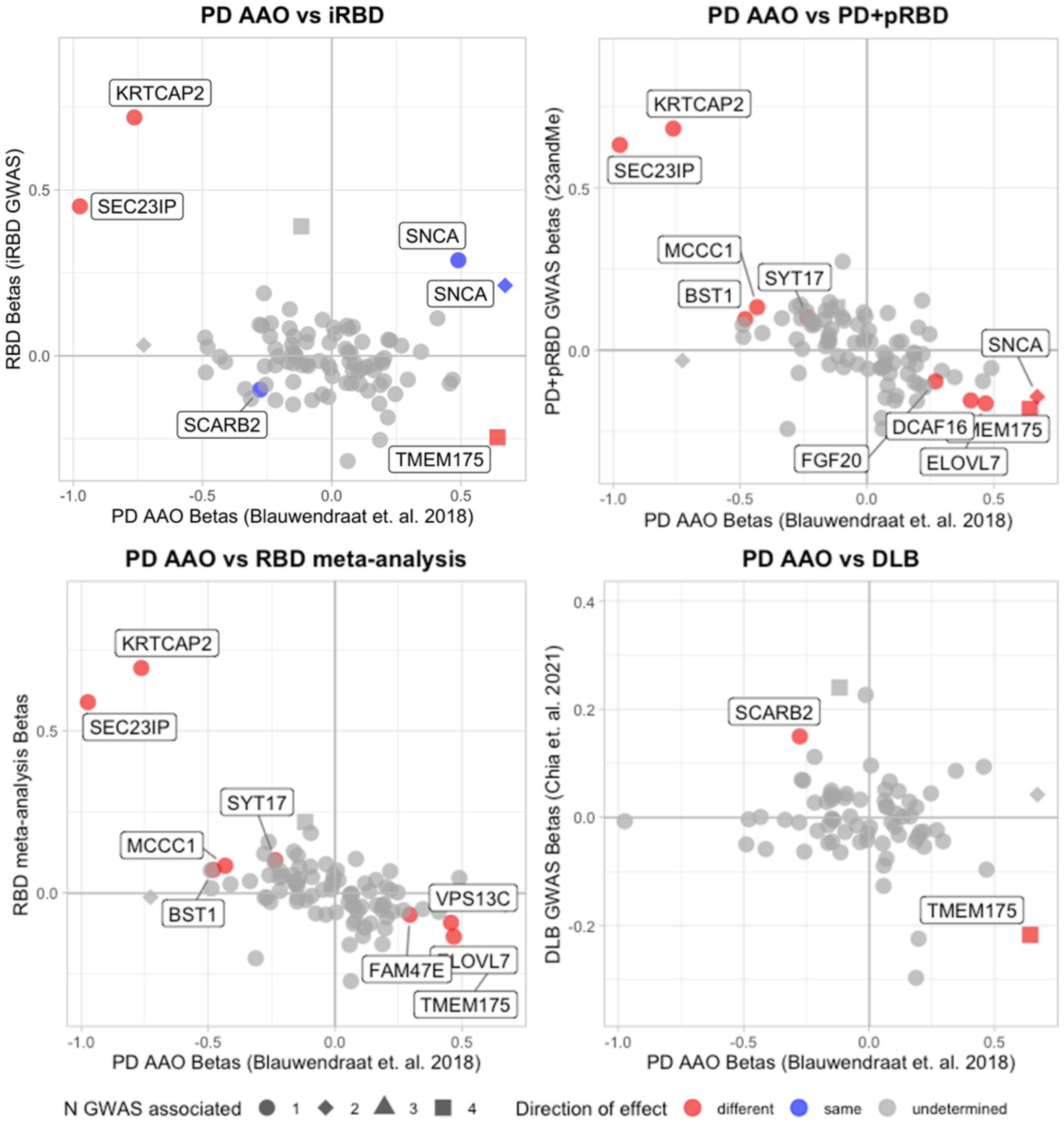

Supplementary Figure 10. Beta-beta plots comparing direction of effect for GWAS-nominated synucleinopathy loci (logistic regression across the genome, case status as dependent variable) and their effects on PD age at onset, as identified by GWAS (linear regression across the genome, age at onset as dependent variable; Blauwendraat et. al. 2018). As expected, most loci that increase risk for any of the synucleinopathies are associated with an earlier AAO, so we see a consistent "different" effect direction. However, in iRBD, we see a few with the opposite effect; for example, the PD SCARB2 variant is nominally associated with decreased risk for RBD (beta=-0.10, unadjusted p=0.045) and an earlier AAO for PD (beta=-0.28, p=0.028). Similarly, the top PD variant rs356182 is concordant in the PD and PD AAO summary statistics but not in iRBD. The allele associated with increased risk for PD (beta=0.28, p=3.9e-154) is associated with earlier PD AAO (beta=-0.67, p=4.6e-08), but shows a potential for the opposite effect in iRBD (beta=-0.23, p=1.5e-04).

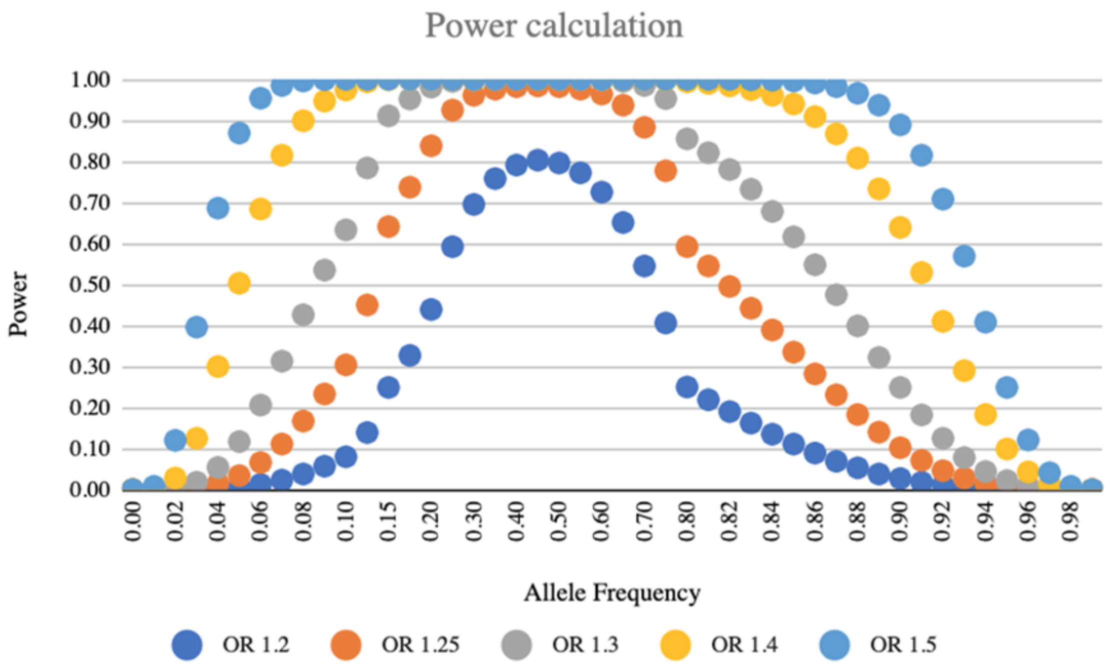

Supplementary Figure 11. Power calculations for different allele frequencies and different odds ratios in the meta-analysis.

SUPPLEMENTARY TABLES

Supplementary Table 1. Replication of meta-analysis variants in an independent PD+pRBD cohort.

SNP	Position (hg19)	Closes gene	EA	Odds ratio (95% CI)	p	Power
rs3756059*	4:90757272	*SNCA*	A	1.22 (1.00-1.48)	0.048	0.70
rs12752133*	1:155205378	*GBA*	T	3.00 (1.02-8.83)	0.047	0.40
rs76763715	1:155205634	*GBA*	C	1.30 (0.05-33.04)	0.888	0.28
rs34311866*	4:951947	*TMEM175*	C	1.28 (1.62-2.04)	0.041	0.40
rs117896735	10:121536327	*INPP5F*	A	0.94 (0.43-2.05)	0.887	0.37
rs7697073	4:77132634	*SCARB2*	T	1.19 (0.97-1.46)	0.090	0.39

SNP: single-nucleotide polymorphism; EA: effect allele; CI: confidence interval. Logistic regression results for meta-analysis GWAS-nominated loci in an independent cohort of PD+pRBD (N=285) and controls (N=907), adjusted for age, sex, and PC1-6. Displayed p-values are unadjusted and two-sided. SNPs with statistical significance ($p<0.05$) are indicated with *. Power to detect the meta-analysis effect size was calculated using the University of Michigan GAS Power Calculator (https://csg.sph.umich.edu/abecasis/gas_power_calculator/index.html).

Supplementary Table 2. Directly genotyped rare variants with previously reported relationships with RBD.

SNP	Gene	Protein change	**% carriers, cases** 23andMe/McGill	**% carriers, controls** 23andMe/McGill	**GT rate** 23andMe/McGill
rs76763715	*GBA*	p.N370S	0.020/0.013	0.007/0.003	>0.99/>0.99
rs34637584	*LRRK2*	p.G2019S	0.007/0.00	0.002/0.0006	>0.99/>0.99

SNP: single-nucleotide polymorphism; GT: genotype.
