# Supplementary material for: Genome-wide association study of REM sleep behavior disorder identifies polygenic risk and brain expression effects
Source: Nat Commun. 2022 Dec 5;13:7496. doi: 10.1038/s41467-022-34732-5 (PMC9722930; doi:10.1038/s41467-022-34732-5)
Supplement: Supplementary file 3 — Description of Additional Supplementary Files [file 41467_2022_34732_MOESM3_ESM.pdf]

Description of Additional Supplementary Files

File Name: Supplementary Data 1
Description: Summary statistics from the RBD genome-wide association study meta-analysis (repeated logistic regression across the genome, adjusted for age, sex, and principal components) for variants used in polygenic risk score analyses. P-values displayed are unadjusted and two-sided.

File Name: Supplementary Data 2
Description Results of colocalization analysis using the RBD GWAS and eQTLs derived from eQTLGen and PsychENCODE.

File Name: Supplementary Data 3
Description Specificity values of *MMRN1* and *SNCA-AS1* in GTEx and AIBS datasets.

File Name: Supplementary Data 4
Description Detailed significant results from gene-set enrichment with WebGestalt. Two-sided p-values are displayed both unadjusted and FDR-adjusted.

File Name: Supplementary Data 5
Description GWAS summary statistics for all GWAS (logistic regression, case status as dependent variable)-nominated synucleinopathy loci in PD, iRBD, PD+pRBD, the RBD meta-analysis, DLB, and PD age at onset GWAS (linear regression, age at onset as dependent variable). P-values are unadjusted and two-sided.

File Name: Supplementary Data 6
Description LD-score regression genetic correlation results for iRBD, PD+pRBD, and the RBD GWAS meta-analysis. Two sided correlation p-values are displayed both unadjusted and FDR-adjusted.
