# Supplementary material for: Genome-wide association study of REM sleep behavior disorder identifies polygenic risk and brain expression effects
Source: Nat Commun. 2022 Dec 5;13:7496. doi: 10.1038/s41467-022-34732-5 (PMC9722930; doi:10.1038/s41467-022-34732-5)
Supplement: Supplementary file 5 — Reporting Summary [file 41467_2022_34732_MOESM5_ESM.pdf]

nature portfolio

Corresponding author(s): Ziv Gan-Or

Last updated by author(s): 2022/11/02

Reporting Summary

Nature Portfolio wishes to improve the reproducibility of the work that we publish. This form provides structure for consistency and transparency in reporting. For further information on Nature Portfolio policies, see our Editorial Policies and the Editorial Policy Checklist.

Statistics

For all statistical analyses, confirm that the following items are present in the figure legend, table legend, main text, or Methods section.

n/a	Confirmed	
☐	☒	The exact sample size (*n*) for each experimental group/condition, given as a discrete number and unit of measurement
☐	☒	A statement on whether measurements were taken from distinct samples or whether the same sample was measured repeatedly
☐	☒	The statistical test(s) used AND whether they are one- or two-sided<br>*Only common tests should be described solely by name; describe more complex techniques in the Methods section.*
☐	☒	A description of all covariates tested
☐	☒	A description of any assumptions or corrections, such as tests of normality and adjustment for multiple comparisons
☐	☒	A full description of the statistical parameters including central tendency (e.g. means) or other basic estimates (e.g. regression coefficient) AND variation (e.g. standard deviation) or associated estimates of uncertainty (e.g. confidence intervals)
☐	☒	For null hypothesis testing, the test statistic (e.g. *F*, *t*, *r*) with confidence intervals, effect sizes, degrees of freedom and *P* value noted<br>*Give P values as exact values whenever suitable.*
☐	☒	For Bayesian analysis, information on the choice of priors and Markov chain Monte Carlo settings
☒	☐	For hierarchical and complex designs, identification of the appropriate level for tests and full reporting of outcomes
☐	☒	Estimates of effect sizes (e.g. Cohen's *d*, Pearson's *r*), indicating how they were calculated

Our web collection on statistics for biologists contains articles on many of the points above.

Software and code

Policy information about availability of computer code

Data collection	No software was used.
Data analysis	For GWAS quality control and analysis, we used PLINK 1.9, R version 4.0.1, GCTA-COJO, rvtests, and METAL. We used PRSice2 and PLINK 1.9 for polygenic risk score calculations, WebGestalt for pathway analysis, Coloc version 4.0.1 (https://github.com/chr1swallace/coloc) for colocalization, with specificity calculated using a modification of published code (https://github.com/jbryois/scRNA_disease and https://github.com/NathanSkene/EWCE). For genetic correlation and heritability, we used the ldsc python package (http://ldsc.broadinstitute.org/ldhub/) on python v2.7. We performed power calculations using the University of Michigan GAS Power Calculator (https://csg.sph.umich.edu/abecasis/gas_power_calculator/index.html). The code used for all analyses are available on https://github.com/lynnekrohn/RBD_GWAS, except for colocalization analyses, which is available on https://github.com/RHReynolds/RBD-GWAS-analysis/. The software used are all pre-established and publicly available; no custom algorithms were implemented.

For manuscripts utilizing custom algorithms or software that are central to the research but not yet described in published literature, software must be made available to editors and reviewers. We strongly encourage code deposition in a community repository (e.g. GitHub). See the Nature Portfolio guidelines for submitting code & software for further information.

Data

Policy information about availability of data

All manuscripts must include a data availability statement. This statement should provide the following information, where applicable:

- Accession codes, unique identifiers, or web links for publicly available datasets
- A description of any restrictions on data availability
- For clinical datasets or third party data, please ensure that the statement adheres to our policy

The iRBD summary statistics are publicly available on GWAS Catalog (study accession GCST90204200). The full GWAS summary statistics for the 23andMe discovery data set will be made available through 23andMe to qualified researchers under an agreement with 23andMe that protects the privacy of the 23andMe participants. Please visit https://research.23andme.com/collaborate/#dataset-access/ for more information and to apply to access the data.
The GWAS summary statistics for traits analyzed for genetic correlation with RBD can be found on LDHub (http://ldsc.broadinstitute.org/ldhub/) or GWAS Catalog (https://www.ebi.ac.uk/gwas/). The quantitative trait loci data used for fine-mapping can be accessed on: eQTLGen https://www.eqtlgen.org/cis-eqtls.html; PsychENCODE http://resource.psychencode.org/; GTEx v8 https://www.gtexportal.org/home/; AIBS https://portal.brain-map.org/atlases-and-data/rnaseq/human-mtg-smart-seq; and Bryois et. al. CNS-specific cell types https://malhotralab.shinyapps.io/brain_cell_type_eqtl/.

Field-specific reporting

Please select the one below that is the best fit for your research. If you are not sure, read the appropriate sections before making your selection.

☒ Life sciences ☐ Behavioural & social sciences ☐ Ecological, evolutionary & environmental sciences

For a reference copy of the document with all sections, see nature.com/documents/nr-reporting-summary-flat.pdf

Life sciences study design

All studies must disclose on these points even when the disclosure is negative.

Sample size	Given the limited availability of REM sleep behavior disorder samples worldwide, due to low awareness and elaborate diagnosis methods, we chose to include all possible samples available in the study.
Data exclusions	Sample exclusion criteria were pre-established based on standardized genome-wide association study procedures. Samples were excluded for non-European ancestry, poor genotype quality, relatedness to other samples closer than cousin, and for differences in genetically determined sex and documented sex (an indicator of sample mismatch).
Replication	Each nominated GWAS locus has been implicated in either REM sleep behavior disorder (SNCA: Bjornara et. al. 2018, Krohn et. al. 2020; GBA: Gan-Or et. al. 2015, Krohn et. al. 2020; TMEM175: Krohn et. al. 2020) or a related alpha-synucleinopathy (INPP5F: Nalls et. al. 2014, Chang et. al. 2017, Nalls. et. al. 2019; SCARB2: Do et. al. 2011, Nalls et. al. 2014, Chang et. al. 2017, Nalls et. al. 2019; GBA: Lwin et. al. 2004, Gan-Or et. al. 2008, Sidransky et. al. 2012, Nalls et. al. 2014, Chia et. al. 2021; SNCA: Polymeropolous et. al. 1997, Singleton et. al. 2003, Mata et. al. 2010, Nalls et. al. 2014, Chia et. al. 2021), so we did not include a large independent replication set. This was also a choice to maximize power given the limited sample availability mentioned in the "sample size" section. All significant variants were at least nominally significant ($p<0.05$) in both cohorts before meta-analysis, and all had the same direction of effect in a small, independent replication PD+pRBD cohort. All rare (minor allele frequency < 0.05) GWAS-nominated variants were validated using Sanger sequencing.
Randomization	All participants that met our criteria for case or control status, age-matching, and quality control were included to maximize power.
Blinding	Individual identifying information was masked for all researchers who handled any genetic data via sample ID (as opposed to participant's name).

Reporting for specific materials, systems and methods

We require information from authors about some types of materials, experimental systems and methods used in many studies. Here, indicate whether each material, system or method listed is relevant to your study. If you are not sure if a list item applies to your research, read the appropriate section before selecting a response.

Materials & experimental systems

n/a	Involved in the study
☒	☐ Antibodies
☒	☐ Eukaryotic cell lines
☒	☐ Palaeontology and archaeology
☒	☐ Animals and other organisms
☐	☒ Human research participants
☒	☐ Clinical data
☒	☐ Dual use research of concern

Methods

n/a	Involved in the study
☒	☐ ChIP-seq
☒	☐ Flow cytometry
☒	☐ MRI-based neuroimaging

Human research participants

Policy information about studies involving human research participants

Population characteristics

Cases and controls in both cohorts have confirmed European ancestry using principal component analysis, and principal components were included as covariates to account for outlying ancestry structure. Controls younger than 45 were not included in analysis as REM sleep behavior disorder is an age-related condition, commonly presenting >50 years of age. Since it is also a male-dominated condition, male controls were favored so all case and control groups were male-dominant (>60%). Sex and age were included as covariates to account for further discrepancies.

Recruitment

For idiopathic RBD (iRBD), cases were selected based on video-polysomngography-confirmed REM sleep behavior disorder. Since this diagnosis requires an overnight consultation in a sleep lab, the cohort may be biased towards individuals with easy access to this kind of health care, and/or more severe cases. For example, those who move forcefully enough to bother themselves or a bed partner will be more likely to seek answers and treatment. PD with probable RBD (pRBD) was identified by questionnaire (RBD Single-Question Screen). Questionnaire-identified patients outside of the Parkinson's population were not included; the questionnaire is not reliable for the general population but much more reliable for Parkinson's patients (sensitivity of 93.8% and specificity of 87.2%). Except for the independent PD+pRBD cohorts from McGill, all questionnaire samples participated in 23andMe. Bias towards those who have been exposed to and can afford this service is present, perhaps skewing the cohort toward a middle- to upper-class demographic. Participants provided informed consent and participated in the research online under a protocol approved by the external AAHRPP-accredited IRB, Ethical & Independent Review Services (E&I Review). Participants were included in the analysis on the basis of consent status as checked at the time data analyses were initiated.

Ethics oversight

AAHRPP-accredited IRB, Ethical & Independent Review Services (E&I Review) by the REB of the Montreal Neurological Institute.

Note that full information on the approval of the study protocol must also be provided in the manuscript.
